# Systematic review of chewing simulators: Reality and reproducibility of *in vitro* studies

**DOI:** 10.4317/jced.57279

**Published:** 2020-12-01

**Authors:** Sergio Soriano-Valero, Juan-Luis Román-Rodriguez, Rubén Agustín-Panadero, Carlos Bellot-Arcís, Antonio Fons-Font, Lucía Fernández-Estevan

**Affiliations:** 1 Private Practice, Alicante, Spain; 2 Prosthodontics and Occlusion Unit, Department of Stomatology, Faculty of Medicine and Dentistry, University of Valencia, 46010 Valencia, Spain; 3 Orthodontics Teaching Unit, Department of Stomatology, Faculty of Medicine and Dentistry, University of Valencia, 46010 Valencia, Spain

## Abstract

**Background:**

The aim of this systematic review was to analyze the types of human chewing simulator described in scientific literature.

**Material and Methods:**

An electronic search was conducted in the databases PubMed, Embase and Scopus. The search strategy included 10 search terms: “*in vitro*”; “dental materials”; “shear strength”; “fatigue fracture”; “bite force”; “prosthetic materials”; “chewing simulator”; “chewing machine”; “simulated mastication”; and “dental wear simulator.” Two researchers worked independently to assess the titles and abstracts of the articles. The quality of the *in vitro* trials selected was evaluated by means of the Consolidated Standards of Reporting Trials scale.

**Results:**

The electronic search identified 80 articles related to the topic of interest. After reading the full texts, ten works were selected. The articles focused mainly on the design of chewing simulators. Most of them were considered of moderate quality. Regarding the characteristics that an ideal chewing simulator should encompass, the devices described in articles varied greatly in terms of movement, periodontal ligament simulation, force sensors, and the materials tested.

**Conclusions:**

No chewing simulator offers all the characteristics necessary to reproduce human masticatory movements and forces under the humidity and pH conditions of the oral cavity. A simulator that encompasses all these characteristics would make it possible to standardize trials involving simulated mastication.

** Key words:**In vitro, dental materials, dental wear simulator.

## Introduction

The mechanical properties of the materials used in dentistry must be thoroughly tested under laboratory conditions before they can enter clinical use. Trials of mechanical resistance (hardness, fracture resistance, elasticity modulus, etc.) must be complemented by wear and fatigue testing.

*Wear* means “to damage, erode, or destroy by friction or use,” while *fatigue* means “weakness in metal or other materials caused by repeated variations of stress.” In dentistry the usual terms used to describe these scenarios are aging and *fatigue* (1).

The need for information about the wear and fatigue characteristics of dental materials before they enter clinical use has led to the development of a range of devices intended to simulate mastication. These simulators are used before final load testing to provide information about a material’s behavior during prolonged use. At the same time, many *in vitro* trials need to imitate, as far as possible, the physiological characteristics of human mastication, and the direction and force of jaw movements (2,3).

The Federal Drug Administration (FDA) has established a series of directives for good laboratory practice. But to date, no single standardized system has been determined for measuring wear and aging, as is the case when measuring traction and fracture resistance. In the currently available chewing simulators, variability in terms of the control and regulation of forces impacts negatively on the reproducibility and variability of results, and the difficulty of extrapolating *in vitro* findings to the oral cavity (4).

The aim of the present systematic review was to analyze the types of chewing simulator described in scientific literature.

## Material and Methods

This systematic review was conducted in accordance with Preferred Reporting Items for Systematic Reviews and Meta-Analyses (PRISMA) (5). It could not be registered in the PROSPERO database as it investigated *in vitro* studies.

The review’s PICO (participants, intervention, comparison, outcome) question was: Which chewing simulator most resembles human mastication, in which P= chewing simulators; I = *in vitro* mastication; C = analysis; and O = human mastication.

The search identified articles that analyzed *in vitro* wear resulting from the action of chewing simulators. Inclusion criteria were: chewing device design was described, and test samples were described (teeth studied/antagonist teeth). These criteria were chosen to center the review on articles involving chewing simulation and oral conditions.

Firstly, an electronic search was conducted in the databases PubMed, Embase and Scopus. The search strategy included 10 search terms: “*in vitro*”; “dental materials”; “shear strength”; “fatigue fracture”; “bite force”; “prosthetic materials”; “chewing simulator”; “chewing machine”; “simulated mastication”; and “dental wear simulator”. Boolean operators (“OR” and “AND”) were applied to link search terms to the research question (Table 1).

**Table 1:**
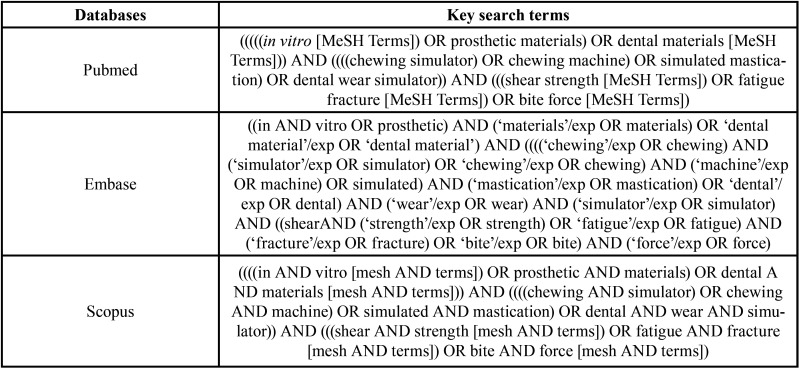
Search strategy used to locate studies in primary electronic databases.

Two reviewers worked independently (L.F.-E. and S.S.-V.) to assess the titles and abstracts of the articles identified in the initial search. The following variables were recorded for each article: author and year of publication, device, study teeth/antagonist teeth, simulated mastication movement, force sensors.

To assess the *in vitro* studies included in analysis, their quality was evaluated by means of the Consolidated Standards of Reporting Trials (CONSORT) scale, modified to assess the quality of *in vitro* trials of dental materials (6). This modified CONSORT scale consists of 15 items that assess the quality of studies in terms of the abstract, introduction, method, results, discussion and other information of interest such as financial support received or access to a description of the test protocol employed. Items included in an article are marked with an asterisk; when a box remains blank this means that the item has not been mentioned in the text.

## Results

**Figure 1:**
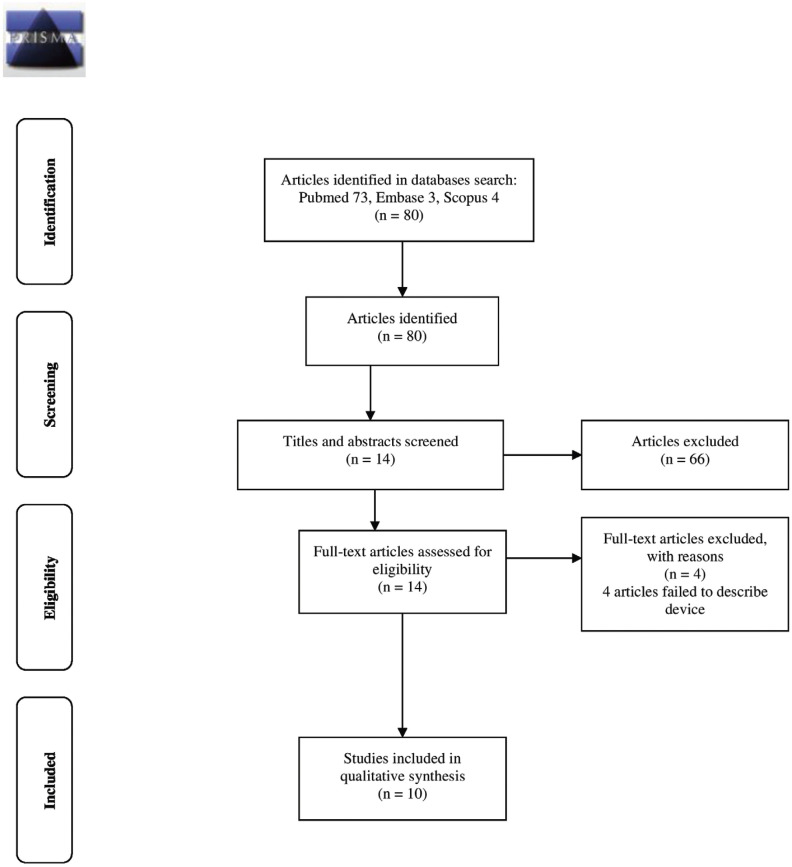
PRISMA 2009 Flow Diagram.

The electronic search identified 80 references related to the research topic, 73 in Pubmed, 3 in Embase and 4 in Scopus. Fifty-seven articles were excluded on the basis of the title, and a further nine after reading the abstracts. After reading the full texts, another four articles were excluded as they failed to provide information relevant to the review (Fig. 1). Finally, 10 articles were selected for review (Table 2), all of which focused to greater or lesser extent on chewing simulator design.

**Table 2:**
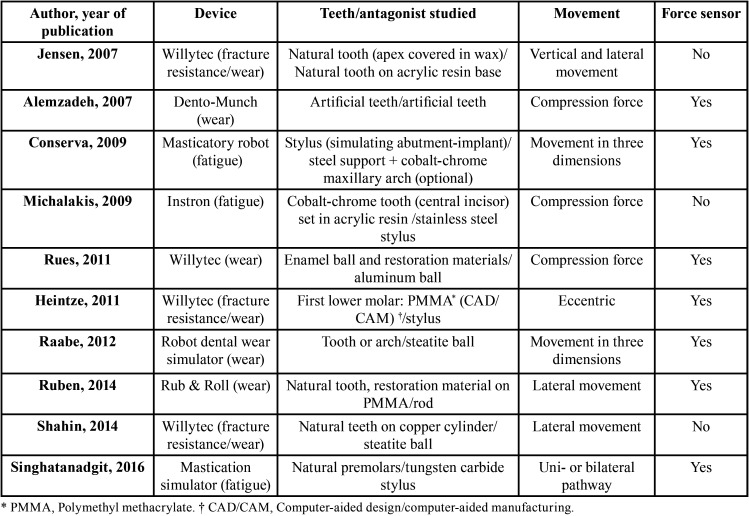
Variables analyzed in systematic review.

The ten articles selected for review were *in vitro* experimental studies. Their quality was evaluated by means of the CONSORT scale. Most of them were considered of moderate quality, as all fulfilled around 8 items out of the total of 15. Table 3 shows the results of the CONSORT scale. All works included a complete abstract (item 1). They also included an introduction that described the study’s scientific antecedents and purpose (item 2a), objectives, and/or hypothesis (item 2b). Regarding the methods section, all explained the intervention carried out on each material evaluated (item 3), and the data recorded in the results, including a description of the test procedure and moment when each material was tested (item 4). The method used to generate a random allocation sequence location and the mechanism used to implement the random allocation sequence (items 6 and 7) were only found in five articles. The statistical methods applied were described in seven works (item 10). For each primary and secondary outcome, results for each group, and the estimated size of the effect and its precision (item 11) were only reported in two articles. All the works suffered trial limitations (item 12). Five articles reported sources of funding. None of the studies explained how the sample size was determined (item 5), who generated the random allocation sequence, who enrolled teeth, who assigned teeth to intervention (item 8), who was blinded and how (item 9), or where the full trial protocol could be accessed (item 14).

**Table 3:**
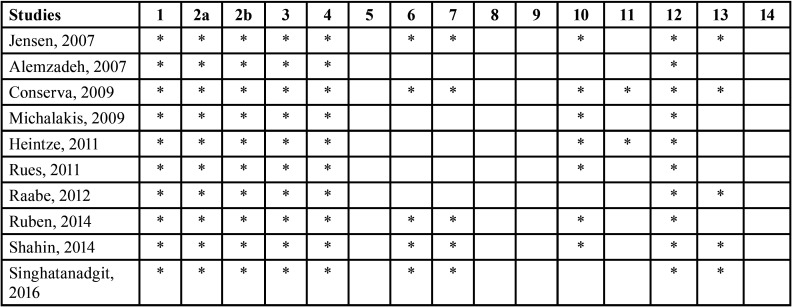
Quality of articles assessed with modified CONSORT scale for in vitro studies. Criteria: 1) Structured abstract; 2a) Scientific antecedents; 2b) Objectives and/or hypothesis; 3) Intervention; 4) Way and moment when outcomes were evaluated; 5) Sample size determination; 6) Method used to generate a random allocation sequence; 7) Mechanism used to generate a random allocation sequence; 8) Who generated random allocation sequence; 9) Who was blinded to random allocation and how; 10) Statistical methods for comparing outcomes; 11) Precision of results obtained; 12) Study limitations; 13) Sources of finance; 14) Access provided to study protocol.

The chewing simulators described were used for fatigue testing, and to measure resistance to fracture and wear. Jensen *et al.* (7), Heintze *et al.* (8), Rues *et al.* (4), and Shahin *et al.* (9), employed the Willytec chewing simulator (SD Mechatronic GmbH, Feldkirchen-Westerham, Germany). It has two motor driven axes to simulate different motion patterns, which are controlled by software that can program all the paths of the masticatory cycle. This device can be used to analyze the wear/fracture resistance of materials used in implants, crowns, bridges, composite restorations, jaw models; the antagonist can be a natural tooth, a carbide or steatite sphere, etc. The simulator is equipped with eight test chambers that can be used simultaneously (although there are smaller models with two or four chambers), each with an individual bar and weight; all the bars are linked by a transversal bar driven by a motor, which ensures that the test conditions are identical in each chamber. It is possible to modify the active axis’s load as well as its sliding motion. The chambers can be filled with water or left unfilled.

The article by Michalakis *et al.* (10) employed a universal fatigue test machine (Instron Corp, Norwood, Mass, USA).

The masticatory robot described by Conserva *et al.* (2,11) consists of two parts, the first being the robot and its control system, while the second records data. The control system is an industrial computer that gives orders to the robot’s moving parts, a Stewart platform, controlling the movements it makes assisted by feedback signals. The Stewart platform is a parallel mechanism formed by an (upper) mobile platform (which simulates the mandible), connected to a fixed base by six identical kinetic jacks, equally spaced, symmetrically arranged to form two equilateral triangles over a fixed base. When the lengths of the jacks are varied by three linear force transmitters, it is possible to change the tilt of the platform through six degrees of freedom (three degrees of freedom for rotation, and three for tilting) replicating the force and motion of functional mastication. Each leg is made up of two steel cylinders each joined to the platform and the base by ball joints at each end. The robot’s motion is determined by the platform position in x, y, and z positions and the platform’s orientation defined as angles to the axes. The x, y, z axes represent the latero-lateral, anteroposterior and vertical axes, respectively. The antagonist is a steel plate, with the option of adding a cobalt-chrome maxilla. This simulator is used to analyze material fatigue.

Singhatanadgit *et al.* (3) developed a simulator designed to generate both unidirectional and bidirectional movements. It consists of two main parts: the upper part replicates the maxilla and the lower simulates the movement of the mandible. It uses a four-bar linkage mechanism to replicate mandibular motion and is used to analyze material fatigue.

Raabe *et al.* (12) employed a simulator consisting of a platform that replicates the static maxilla or any antagonist. The (lower) mobile portion impacts on the static part by means of six propelling arms which, in combination, simulate mandibular movements of variable force. The lower platform is equipped with a resilient system that imitates periodontal ligament. It also has a wet chamber that can be used with water, artificial saliva or other liquids.

Ruben *et al.* (13) proposed a machine – they called it “Rub & Roll” – for wear-testing materials placed in a gyrating cylinder that generates a rubbing action between a material inserted in the cylinder and a static surface, which can be of any type of material.

Alemzadeh *et al.* (14) employed a device they called “Dento-Munch.” This consists of a fixed base (lower plate) that does not move and platform (upper plate) which is set parallel to the lower plate and moves with six degrees of freedom varying the length of one or more of the force transmitters; this was used to study wear to materials.

Regarding the materials used both for test teeth/structures and antagonist teeth/structures, of the 10 articles reviewed, three used natural teeth as the test object (3,7,9), one used a simulated implant-abutment set-up (11), one employed cobalt-chrome teeth to which metal-ceramic crowns were cemented (10), one used standardized (CAD/CAM) PMMA mandibular molars (8), one involved different sample types (enamel or dentin discs, or natural molar-shaped samples, teeth with or without roots, restoration materials, etc.) (13) one used discs of enamel and of restoration materials (4), another used artificial teeth consisting of a resin crown retained on a ceramic root by universal resin cement (14), and one used universal resin composite as a material for reproducing dental specimens (12).

As for the antagonist teeth used in the papers reviewed, only one article used natural teeth set in an acrylic base (14). The other works used steel, tungsten carbide, aluminum or steatite as antagonist materials: one article used a steel element with the option of adding a cobalt-chrome maxillary arch (11), two used a stainless steel stylus (8,10), one work used a PVC-coated stainless steel stylus (13), one used a tungsten carbide stylus (3), one used an aluminum sphere (4), and two a steatite ball (9,12).

Various materials were used to simulate the behavior of periodontal ligament, aiming to imitate its resilience in force transmission. One study employed wax to replicate periodontal ligament (7), another silicon (9), while two used rubber (13,14). A load cell was used in one study, placed below the static antagonist to imitate the feedback from periodontal proprioceptors (12). In the study by Michalakis *et al.* (10), cobalt-chrome teeth were set in self-curing acrylic resin blocks with the block surface placed 2 mm below the crown’s margin. This design simulated the clinical conditions of a healthy periodontium. In the four remaining articles, no simulation of periodontal ligament was mentioned (3,4,8,11).

In addition to the chewing simulator devices and the samples tested in them, it is important to analyze the movements made by the antagonist structures. In three studies, the devices generated a simulation of masticatory movement and masticatory forces in three dimensions (3,11,12). In another three articles, the chewing simulators applied vertical compression forces exclusively (4,10,14). Another simulator applied eccentric or lateral forces (13). In the three remaining studies, the simulators employed vertical and lateral movements but not three-dimensional motion (7-9).

In addition to the movements produced by the chewing simulator devices, the present review also analyzed the use (or absence) of load sensors for measuring the forces exerted. Seven articles stated the force applied. Conserva *et al.* (11) used a base equipped with a sensor fixed to the robot’s mobile platform (mandible), which recorded the degree of force transmitted through the axes (x, y, and z). Heintze *et al.* (8) employed a 3D piezoelectric force sensor during dynamic loading; this was fixed to a special support at the lower end of the vertical load bar. Ruben *et al.* (13) used a single point load cell on the outside of the container equipped with a measuring sensor. Singhatanadgit *et al.* (3) employed a miniature load cell between the tungsten carbide stylus and the base of the mobile weight. Rues *et al.* (4) considered that, if there was only one force sensor available, then the eight chambers should be measured separately. Alemzadeh *et al.* (14) used extensiometric strain gauge force transducers on the mandible below the second molars to measure axial bite forces applied to posterior teeth. Raabe *et al.* (12) placed a load cell below the static antagonist. The three remaining works made no mention of having employed any force sensor (7,9,10).

## Discussion

In recent years, various chewing simulators have attempted to reproduce the oral environment in order to test dental materials under conditions as close as possible to *in vivo* conditions (15).

On the basis of the present review findings, the ideal chewing simulator should have specimen positioning devices that simulate human jaws. Among the articles reviewed, only one study placed natural teeth against the same type of antagonist, although this consisted of single teeth rather than complete arches (7). The literature does not describe any device that uses complete upper and lower jaws with natural teeth for wear testing.

The natural teeth used in these devices must be set on structures with similar characteristics to natural supporting structures (periodontal ligament and alveolar bone), made from materials that exhibit resilient rather than rigid behavior. In this context, silicon and acrylic resin have been used to imitate periodontal ligament and alveolar bone, respectively (16).

The simulator must be able to generate movements that imitate human mastication as far as a machine is able to. In mastication, the mandible moves in vertical and horizontal direction, making a bidirectional motion (3,17). However, most of the devices fail to reproduce the complex movements of mastication in all three dimensions (2). The ideal simulator should be able to make opening and closing movements, as well as eccentric (lateral and protrusive) movements in order to analyze wear to materials subjected to the vertical and horizontal loads that occur in mastication motion. Although an ideal simulator does not yet exist, many of the simulators described above are able to generate motion that is not limited to compression alone (3,7-9,11-13).

If wear testing is to be standardized and obtain comparable results, it is essential for the magnitude of forces generated by chewing simulators to be controlled to create specific test conditions, and for these to be identical for each specimen tested. For this reason, the simulator must be equipped with load sensors to record the loads applied, as in some of the studies reviewed (3,4,8,11-14). It is important to know the exact load applied to each dental group, as this will differ from group to group.

To simulate conditions in the oral medium, some studies have attended to environmental factors such as humidity/wetness, temperature and pH, which can also influence the mechanical properties and behavior of dental materials in the mouth. The body acts as a heat pump that maintains the temperature in the oral cavity at 37ºC. Teeth and any restoration material are continuously bathed in saliva with a pH of around 7. Humidity in the cavity is 100%. However, the introduction of different foods in the mouth can alter environmental conditions to a large degree, causing fluctuations in pH and temperature. An artificial oral environment should be able to reproduce normal conditions in the oral cavity, and to manage the temperature and pH fluctuations that may occur. *In vitro* studies may use human saliva or de-ionized water, among other liquids, as a lubricant or even as an abrasive/erosive medium (3,12,13,15,18).

There are few bibliographic references regarding the design of models for use in chewing simulators for trials involving simulated mastication. Several parameters have not been sufficiently investigated, including the curve of Spee and the curve of Wilson. Alemzadeh *et al.* (14) analyzed the curve of Spee digitally. Another relevant parameter is the angle of the occlusal plane at which maxillary models are set in the simulator; no research has contemplated this parameter in terms of replicating masticatory movements. It is necessary to standardize the values of these parameters in order to analyze wear to dental materials in models with identical characteristics.

The present systematic review suffered certain limitations. Despite conducting a search in the databases, only a small number of relevant articles were identified, which might be explained by the difficulties involved in carrying out the type of *in vitro* study that was the subject of the review. The search did not include the Cochrane database as it does not admit *in vitro* research. The quality of most of the studies was moderate as it is hard for this type of *in vitro* research to meet the criteria that would deem it of high quality.

## Conclusions

The present systematic review did not identify any chewing simulator that includes all the characteristics that would make it possible for complete upper and lower dental arches to reproduce the movements of human mastication under the actual humidity and pH conditions in the oral cavity. There is need for a simulator that meets all these requirements in order to standardize future trials involving simulated mastication.
